# Use of the Composite Pedicled Pectoralis Minor Flap after Resection of Soft Tissue Sarcoma in Reconstruction of the Glenohumeral Joint

**DOI:** 10.1155/2014/937342

**Published:** 2014-12-31

**Authors:** Michiel A. J. van de Sande, Tom Cosker, Stephen M. McDonnell, C. L. M. H. Gibbons, Henk Giele

**Affiliations:** ^1^Department of Orthopaedics, LUMC, Albinusdreef 2, 2300 RC Leiden, Netherlands; ^2^The Oxford Bone and Soft Tissue Tumour Service, Nuffield Orthopaedic Centre, Oxford, UK

## Abstract

The surgical repair of an extensive anterior glenohumeral soft tissue defect is complicated by glenohumeral instability and subsequent significant functional deficit. This surgical note offers a relatively simple reconstruction of the anterior capsule and subscapularis muscle using a pectoralis minor pedicle flap. This reconstruction is supplemented with functional reconstruction of the anterior glenohumeral joint. A conventional deltopectoral approach is utilized and pectoralis minor is freed from its coracoid insertion, released, and mobilized without compromising the pedicle entering from the dorsum and inferior one-third of the muscle. The mobilized pectoralis minor vascular pedicle has sufficient length for the pectoralis minor to be transferred to provide coverage of the anterior shoulder joint even in full external rotation, providing anterior stability. To further improve glenohumeral stability and shoulder function, the pectoralis major muscle can be split with the clavicular part reinserted lateral to the bicipital groove onto the lesser tuberosity replacing subscapularis function while stabilising the glenohumeral joint.

## 1. Introduction

The use of a pectoralis minor pedicle flap has primarily been described for anterior shoulder and breast reconstruction as well as for treatment of unilateral facial palsy [1–4]. Wirth and Rockwood Jr. have described its application for shoulder stabilization in 1997 by transferring the pectoralis minor tendon's insertion from the coracoid process to the greater tuberosity. They concluded this transfer alone was insufficient for subscapularis tear repair [5]. Several tendon transfers or grafts including hamstring, partial subscapularis, long head of biceps, and iliotibial band have also been used to some extent to provide additional stability to the glenohumeral joint [6, 7]. To our knowledge the use of a bipolar pectoralis minor pedicle flap to reconstruct both the anterior glenohumeral capsule and subscapularis tendon has not been previously described.

This surgical note offers a relatively simple reconstruction of the anterior capsule and subscapularis muscle using a pectoralis minor pedicle flap.

## 2. Anatomy

**Figure 1:**
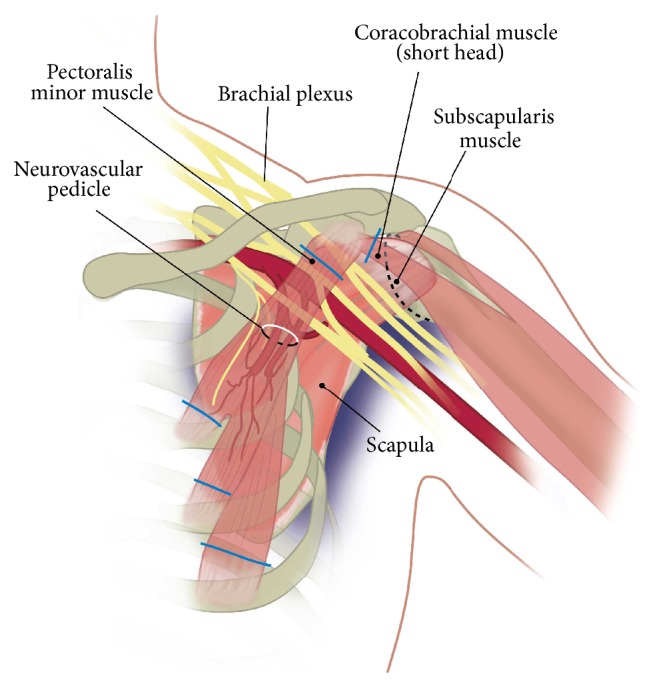
Picture of pectoralis minor origin and insertion and its relation to axillary artery and brachial plexus and penetration point of pedicle.

The pectoralis minor muscle is described as a flat, triangular muscle of the anterior chest wall originating from the 3rd, 4th, and 5th ribs and inserting onto the coracoid process of the scapula (Figure 1). Its neurovascular bundle, originating from the medial pectoral nerve and either one of the lateral thoracic artery (33%), the axillary artery (40%), or the thoracoacromial artery (20%), is situated in the lateral one-third of the muscle on its deep aspect [1]. Its function is as an accessory muscle during inspiration and in scapulothoracic protraction stabilising the scapula during forward flexion in the glenohumeral joint.

Bourdais et al. recently performed an anatomical study to assess anatomical and surgical possibilities of a pectoralis minor pedicle flap around the anterior shoulder [1]. This study concluded that this flap is simple to perform, is reproducible, and should be a primary choice for coverage following tumour resection around the clavicular region.

## 3. Case Report

**Figure 2:**
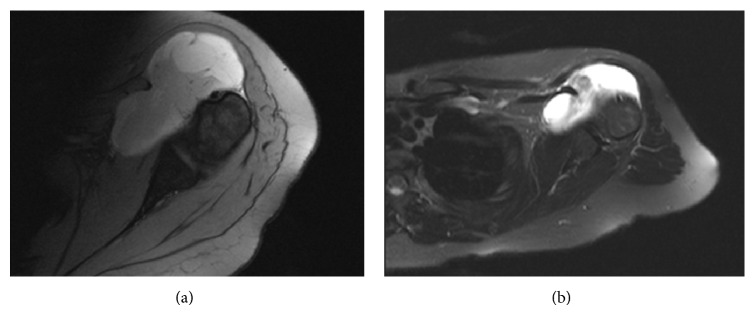
(a) Pre- and (b) postradiation therapy MRI of synoviosarcoma of the anterior shoulder joint.

An 18-year-old student and gifted violinist presented to our bone and soft tissue sarcoma service with a soft tissue sarcoma (high grade synovial sarcoma) of the shoulder (Figure 2(a)). Due to the involvement of the brachial plexus, the size of the tumour, and histological diagnosis, it was initially thought that a forequarter amputation or endoprosthetic replacement would be the only option. A course of neoadjuvant radiation therapy was administered. She received 50 Gray in 25 fractions in 5-week course. The postradiotherapy MRI four weeks later revealed some reduction in tumour size and presented the possibility of primary resection of the tumour taking the subscapularis tendon, anterior capsule, and both heads of biceps but leaving the brachial plexus unharmed (Figure 2(b)).

## 4. Surgical Resection

A deltopectoral approach was employed and wide resection was undertaken. Pectoralis major was detached from the humeral shaft lifting it off the synovial sarcoma leaving a thin layer of muscle and pseudocapsule around it. The short head of biceps and the pectoralis minor were then released from their insertion on the coracoid process to allow better exposure of the brachial plexus which could then be dissected and freed medial to the tumour. The short head of biceps was left attached to the anteromedial tumour border. The superior and inferior borders of subscapularis muscle were identified and the lateral one third of subscapulais muscle and tendon were resected, still fixed to the anteromedial portion of the tumour. The deep aspect of the tumour was then released by careful dissection deep to the posterior aspect of the subscapularis and joint capsule towards its insertion onto the lesser tuberosity that was released subperiosteally. The long head of biceps was cut just above the bicipital grove leaving the distal part with the tumour. There was no free margin between the tumour and the anterior capsule; therefore, it was cut from the anterior labrum leaving the anterior glenohumeral ligament attached to the posterior border of the tumour. Care was taken to ensure that the axillary nerve running at the inferior border of the glenoid was not disrupted. The inner fascia of the deltoid was then dissected and left attached to the lateral border of the tumour. The excised specimen was then sent for histopathological analysis. Histology revealed a high grade synovial sarcoma with intermediate reaction to radiation therapy, resected with disease-free margins, the closest margin being 1 mm at the joint capsule.

## 5. Surgical Reconstruction: Pectoralis Minor Pedicle Flap

**Figure 3:**
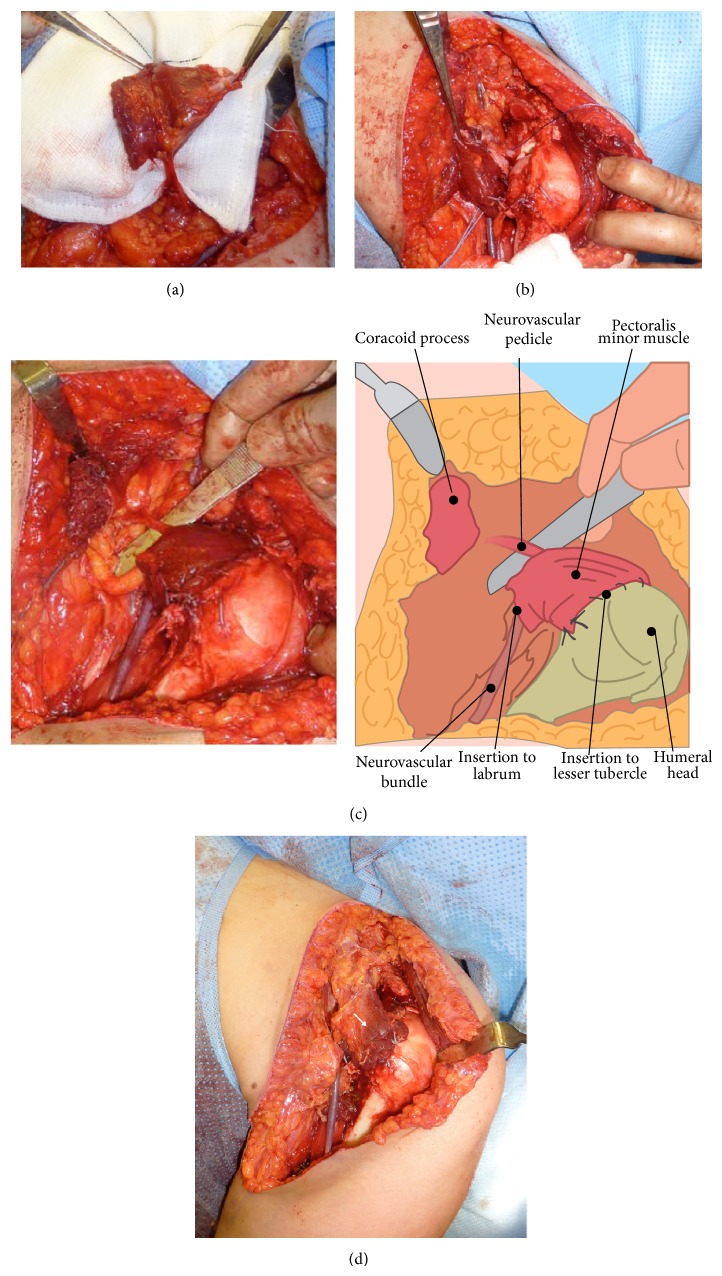
Surgical pictures of pectoralis minor pedicle flap transfer onto the anterior defect of glenohumeral joint capsule. (a) Mobile pedicle flap. (b) Insertion of the pedicle flap onto the anterior labrum and glenoid, covering the sizable defect of the anterior capsule. (c) Fixation of pedicle flap of pectoralis minor onto the lesser tuberosity, showing no tension on the pedicle. (d) Covered glenohumeral joint with pectoralis minor and overlying transferred split pectoralis major muscle to the medial part of greater tuberosity (arrow).

The pectoralis minor tendon lies beneath the pectoralis major muscle and can be carefully dissected and released from its insertion onto the coracoid process and freed from its surrounding tissues leaving the neurovascular bundle situated dorsally at the inferior margin of the musculotendinous junction (Figure 3). The muscle fibres are then released from their insertion on the ribs ensuring a sufficient flap to cover the defect. The neurovascular bundle can be freed from its surrounding tissues in order to provide enough length to transfer the flap of the pectoralis minor to the anterior part of the shoulder joint (Figure 3(a)). To ensure adequate capsular tensioning and provide enough range of movement, the shoulder is placed in 30° of abduction, 30° of external rotation, and 20° of forward flexion prior to capsular reconstruction [6]. The flap is first attached with nonresorbable sutures to the anterior labrum running from the insertion of long head of biceps to the inferior border of the glenoid and is then secured with sufficient tension onto the bicipital groove using bone tunnels.

In this case the pectoralis major muscle was split, leaving the inferior part attached to the humerus, in order to transfer the clavicular part to the lesser tuberosity to function as the subscapularis muscle [8]. In addition, the latissimus dorsi tendon was transferred to the greater tuberosity to provide additional anterior stability and enable it to prevent proximal migration of the humeral head [9].

## 6. Follow-Up

After surgery, she was kept in a shoulder immobilizer for two weeks and a sling for four weeks during which she was slowly mobilized using closed chain movement physiotherapy. Range of motion was slowly increased after 6 weeks. There were no wound healing problems or signs of infection. Now, 40 months after surgery, she has pain-free movement of the shoulder apart from when she fully extends her shoulder and elbow together producing mild median nerve traction pain and paraesthetic symptoms in her thumb and index finger. Nerve conduction studies suggest a radiation-induced plexopathy. She can internally rotate her shoulder fully and externally rotate it to 45 degrees with good power. Active shoulder abduction is possible up to 45 degrees. Passively, it is possible to abduct her shoulder to about 80 degrees. Shoulder active flexion is comparably better at 100°. She can extend and flex her elbow fully and has normal pronosupination and wrist and hand movement. She is using her hand for keyboarding and eating without any problems. There is no sign nor sensation of instability.

**Figure 4:**
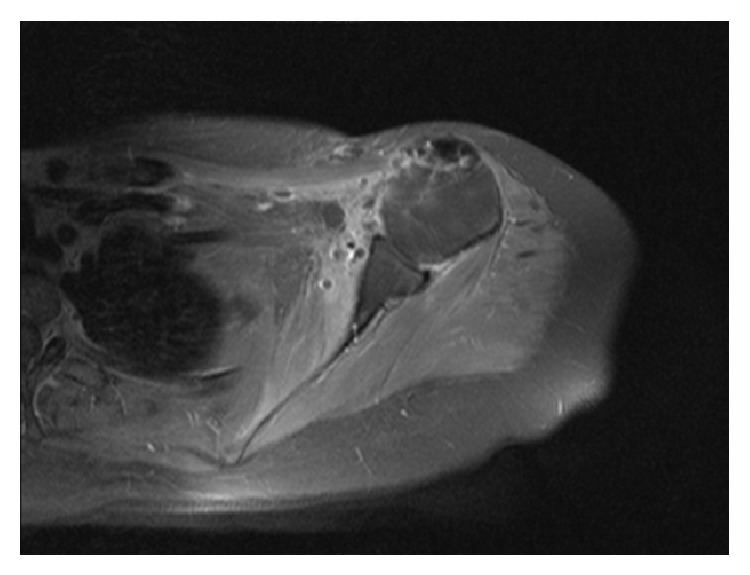
Postoperative MRI showing the anterior shoulder reconstruction with viable pedicle flap of pectoralis minor.

MRI of the shoulder revealed a good central position of the humeral head to the glenoid and no signs of local recurrence (Figure 4). Chest-CT was clear of metastasis.

## 7. Discussion

Surgical repair for anterior glenohumeral soft tissue defects is challenging and complicated by the loss of capsular tissue, including the anterior glenohumeral ligaments as well as the subscapularis tendon. Successful reconstruction using either primary closure aided by extracellular matrix materials or allo- or autograft reconstruction or muscle/tendon transfers using the superior part of the pectoralis major muscle has been reported [5, 7, 10, 11]. Direct surgical repair of the defect could result in functional deficit, as external rotation would be limited and anterior stability would be impaired. The use of an extracellular matrix has been proposed to act as a scaffold to decrease tension on the repaired tissues and aid biological repair but the appropriate clinical applications have not yet been fully defined, and complications such as inflammatory response, graft failure, and infection have been reported [10]. The use of allo- or autograft tendinous reconstruction can effectively provide stability compensation for the insufficient or absent anterior glenohumeral ligaments but, besides donor site morbidity, may be also prone to complications mentioned above [7]. The importance of this reconstructive technique was underlined by Paladini et al. who reported promising results of pectoralis minor transfers in treating upper subscapularis lesions in 27 patients [11].

Finally, improving glenohumeral stability by use of one of many pectoralis major tendon transfers improves stability and range of movement in chronic degenerative rotator cuff disease but is generally not sufficient when additional degenerative cuff pathology is present [8, 12, 13].

The additional transfer of latissimus dorsi tendon to the greater tuberosity can compensate for the loss of external rotation and prevents proximal migration of the humeral head. This transfer is frequently used in massive rotator cuff tear patients suffering from shoulder joint instability and severe functional deficit [9, 14]. In this case, the combined techniques of a pectoralis minor pedicle flap transfer with transfer of pectoralis major and latissimus dorsi have resulted in a stable, pain-free, and functional glenohumeral joint after tumour resection at the anterior shoulder joint. This is in contrast to Omid and Lee who concluded that latissimus dorsi transfer is relatively contraindicated with relative insufficiency of subscapularis [15]. However, in our case, the anterior stability was restored using the pectoralis minor flap and could be used to provide additional stability and rotation.

Combination of radiotherapy, wide surgical resection, and reconstructive shoulder surgery with a vascularized pedicle flap, involving a team of specialists, has provided this young patient with a possible curative surgical resection and a stable and functional shoulder joint. This demonstrates the importance of a specialized bone and soft tissue service combining the knowledge of all involved specialties including pathology, radiology, radiation oncology, and oncology general-, orthopaedic-, and plastic surgery.

## 8. Conclusion

When a defect in the anterior capsule or subscapularis muscle is present, either by surgical resection of soft tissue tumour or as result of a chronic retracted subscapularis tear, the pectoralis minor muscle can be used as a bipolar musculotendinous pedicle flap, in order to close the anterior defect of the glenohumeral joint capsule. It provides enough length of the pedicle to ensure full abduction and external rotation and some functional activity to assist internal rotation. Combined with tendon transfer of the pectoralis major and latissimus dorsi, glenohumeral stability was provided and proved sufficient in this case. In a quickly aging society, this pedicle flap may present a new surgical repair in the armamentarium of the shoulder surgeon especially in chronic instability and subscapularis tendon tear cases.
